# REVERSE TRANSLATION OF HUMAN BIO-AGING MEASURES TO CYNOMOLGUS MONKEYS TO TEST ASSOCIATIONS WITH DOMINANCE RANK

**DOI:** 10.1093/geroni/igz038.1263

**Published:** 2019-11-08

**Authors:** Noah Snyder-Mackler, Carol Shively, Tom Register, Daniel W Belsky

**Affiliations:** 1 University of Washington, Seattle, Washington, United States; 2 Wake Forest University, Winston-Salem, North Carolina, United States; 3 Columbia University Mailman School of Public Health, New York, New York, United States

## Abstract

Social status is a powerful correlate of aging-related health decline. Observational data in humans suggest that disadvantaged social status may be associated with accelerated biological aging. But establishing causality in this relationship poses challenges; experimental manipulation of human social status is not possible. In contrast, social status can be experimentally manipulated non-human primates (e.g. Snyder-Mackler 2016 Science). We conducted analysis to reverse-translate blood-chemistry of biological aging to cynomolgus monkeys using data from several hundred animals in the Wake Primate Center breeding colony. We are applying these measures in an independent sample of monkeys with ascertained dominance rank to test replication in the non-human primate model of the human social gradient in biological aging. Parallel analysis of DNA methylation-based measures of biological aging are ongoing and should be available to present by Fall 2019. Results will inform potential to use this non-human primate model to study social determinants of biological aging.

